# Therapy of intracellular *Staphylococcus aureus* by tigecyclin

**DOI:** 10.1186/1471-2334-13-267

**Published:** 2013-06-05

**Authors:** Carolin A Kreis, Michael J Raschke, Steffen B Roßlenbroich, Nancy Tholema-Hans, Bettina Löffler, Thomas Fuchs

**Affiliations:** 1 Department of Trauma, Hand and Reconstructive Surgery, University Hospital of Muenster, Waldeyerstr. 1, 48149, Muenster, Germany; 2 Institute of Medical Microbiology, University Hospital of Muenster, Domagkstr. 10, 48149, Muenster, Germany

**Keywords:** Osteomyelitis, Implant associated infection, *Staphylococcus aureus*, Tigecyclin, Biomechanical stability

## Abstract

**Background:**

In the fields of traumatology and orthopaedics staphylococci are the most frequently isolated pathogens. *Staphylococcus aureus* and *Staphylococcus epidermidis* are known to be the major causative agents of osteomyelitis. The increasing number of multiresistant *Staphylococcus aureus* and resistant coagulase-negative staphylococci as a trigger of complicated osteomyelitis and implant-associated infections is a major problem. Antibiotic therapy fails in 20% of cases. Therefore the development of novel antibiotics becomes necessary.

**Methods:**

This study analyses tigecyclin, the first antibiotic of the glycylines, as a potential therapy for osteomyelitis caused by multiresistant *Staphylococcus aureus*. Therefore its intracellular activity and the potential use in polymethylmetacrylate-bone cement are examined. The intracellular activity of tigecyclin is determined by a human osteoblast infection model. The investigation of the biomechanical characteristics is conducted concerning the ISO 5833-guidelines.

**Results:**

Tigecyclin shows in vitro an intracellular activity that ranges between the antimicrobial activity of gentamicin and rifampicin. A significant negative effect on the biomechanical characteristics with an impaired stability is detected after adding tigecyclin to polymethylmetacrylate-bone cement with a percentage of 1.225% per weight.

**Conclusions:**

This study shows that tigecyclin might be a potent alternative for the systemic therapy of osteomyelitis and implant-associated infections whereas the local application has to be reconsidered individually.

## Background

Osteomyelitis is an inflammatory process caused by infective microorganism [1]. With a percentage of 80% [2]*S*. *aureus* (*Staphylococcus aureus*) is the most common pathogen of osteomyelitis [1,3]. Implant-associated infections are also often caused by *S*. *epidermidis* (*Staphylococcus epidermidis*) [3,4] and occur with a rate from 3-33% [5]. The therapy of osteomyelitis still represents a major challenge [3]. A single antibiotic agent is the standard therapy for osteomyelitis in its early stages [1]. In order to cure the chronic stages of this bone infection a combined therapy has to be chosen, which consists of a combination of two antibiotic substances and the surgical approach, which implies the surgical debridement, the implant removal or the two-step replacement [1,3,6,7]. The combination of rifampicin plus another antibiotic agent shows efficient therapeutic results for the implant-associated infection caused by *S*. *aureus*[1]. However, antibiotic therapy fails in 20% of cases. This might be explained by the ability of *S*. *aureus* to invade human osteoblast cells [1-3] and to survive intracellularly [1,2]. Thereby, *S*. *aureus* can change its phenotype into SCVs (small colony variants), which is a bacterial subpopulation that is well adapted for long-term intracellular persistence [3,8]. In general SCVs have a reduced metabolism and have an increased resistance against antibiotics, especially against aminoglycosides [3,8,9]. Furthermore, *S*. *aureus* and *S*. *epidermidis* can adhere to foreign material surfaces and form biofilm [1,3,7,10,11]. The bacterial biofilm is a matrix out of polysaccharides and proteins [10] and acts as a diffusion barrier that inhibits the diagnosis and eradication of bacteria [7]. PMMA (polymethylmetacrylate)-bone cement loaded with antibiotic substances is an established treatment for infected endoprotheses for over 40years with good long-term results [6,12]. Antibiotic-loaded bone cement works as a local antibiotic-carrier with the function of a drug-delivery-system to achieve high antibiotic levels locally [12-14] and to decrease the infection rate [12]. PMMA-bone cement is most commonly loaded with aminoglycosides [15], especially with gentamicin as a good prophylaxis as well as a treatment for implant-associated infections [16]. It was shown that PMMA-bone cement loaded with gentamicin inhibits 80% of *S*. *aureus*-biofilm synthesis [17]. Despite these options the treatment of osteomyelitis remains a challenging problem [3]. MRSA- and VRSA- infections are increasing as well as the number of antibiotic-resistant *S*. *aureus* and *S*. *epidermidis*[3,12]. Because of increasing resistance against gentamicin [12,18] and vancomycin [18] new [16] or combined [12] antibiotics have to be added to bone cement. Furthermore 41% of clinical isolated *S*. *aureus* are resistant against gentamicin [16]. *S*. *aureus* also has the ability to grow on bone cement loaded with gentamicin [19]. *S*. *epidermidis* is able to adhere to bone cement in vivo [20] and in vitro and to synthesize biofilm on bone cement loaded with gentamicin [21]. Multiresistant pathogens are a major therapeutic problem [22]. Therefore new antibiotics are essential to minimize pathogens and to optimize the therapy of osteomyelitis [23]. In this context tigecyclin has been developed [24], which is the first antibiotic of the glycylines with efficacy against many important pathogens, multiresistant strains, mixed infections and problematic pathogens [23,24]. In clinical daily routine tigecyclin shows good therapeutic efficacy against intraabdominal infection, skin- and soft-tissue infections [23,24]. Tigecyclin shows activity against infection caused by MRSA [25] and against biofilm associated pathogens [26]. Furthermore tigecyclin is absorbed by bone tissue after intravenous application [27]. Till 2007 only few tigecyclin-resistant *S*. *aureus*-strains were known [28], there are even some rifampicin-resistant *S*. *epidermidis*-strains that were sensitive against tigecyclin [4]. This study investigates tigecyclin as a potential therapy for osteomyelitis caused by multiresistant *S*. *aureus*. Its intracellular activity is determined in comparison to gentamicin and rifampicin by a human osteoblast infection model [29]. The potential use in PMMA-bone cement is conducted concerning the ISO 5833-guidelines compared to gentamicin (International Organization for Standardization: Implants for surgery - Acrylic resin cements. 2002).

## Methods

### Human osteoblast infection model

To determine the intracellular activity of tigecyclin a primary human osteoblast model modified by Haslinger-Loeffler et al. [29] is established. Due to the capacity of *S*. *aureus* to enter the intracellular environment two different *S*. *aureus*-strains (*S*. *aureus* Cowan I ATCC 12598, *S*. *aureus* Subspezies Rosenbach ATCC 49230) with different invasion characteristics (Cowan I: 100%, ATCC 49230: 180%) are used. *S*. *carnosus* TM 300 allegorizes the negative control without capacity to enter the intracellular environment. On the previous day of the assay the human osteoblast cells are seeded together with 1ml growth medium into 12-well tissue culture plates with a number of 1×10^5^/well. The growth medium consists of Minimum Essential Medium (MEM), HAM’s F12 (MEM:HAM’s F12 = 1:1), 10% fetal calf serum (FCS), penicillin/streptomycin (penicillin 100 Units/ml, streptomycin 100 mg/ml), 10nM Dexamethason, 0,2 mML-Ascorbat-2-Phosphate and 10 mM b-Glycerolphosphate. Prior to the investigation the bacteria strains have to be grown overnight. Bacterial cell numbers are measured spectrophotometrically at 540 nm and diluted with 1ml phosphate-buffered saline, pH7.4 (PBS) and with 1% HSA to ensure a cell number of 5×10^8^ bacteria/ml. At the beginning of the assay the confluent grown osteoblast cells are washed with MEM/HAM’s F12 (1:1) and then incubated with the cultured bacteria and 1ml assay medium, which consists of MEM/HAM’s F12 (1:1) and 1% HSA, for 30 min at ambient temperature followed by 3h at 37°C and 5% CO_2_. In order to assure a multiplicity of infection (MOI) of 100 with an optical density (OD) of 1, bacteria are added with the following amounts: *S*. *carnosus* TM 300: 38 μl, *S*. *aureus* Cowan I ATCC 12598: 19 μl, *S*. *aureus* Subspezies Rosenbach ATCC 49230: 16 μl. After incubation the external *S*. *aureus* are inactivated by 20 mg/ml lysostaphin. Then 1ml MEM/HAM’s F12 (1:1) + 10% FCS is added to the osteoblast cells with the intracellular persisting bacteria. For the following incubation for 20h or 40h at 37°C and 5% CO_2_ 10 μg/ml tigecyclin, 10 μg/ml gentamicin, 7 μg/ml rifampicin or the combinations tigecyclin/gentamicin or tigecyclin/rifampicin are added. The antibiotic concentrations were chosen according to high level doses that can be reached after intravenous application in serum and tissue [31-33]. Both *S*. *aureus* strains were tested sensitive to the antibiotics tigecyclin, gentamicin and rifampicin (Table 1). To determine the internalization and the success of the antibiotics the cells are detached from the wells after 20 h or 40 h and washed consistently with PBS, so that the osteoblast cells are destroyed and only the intracellular bacteria are able to remain. Bacteria are resuspended with 1ml aqua dest and cell enumeration of colony forming units (CFU) is performed by serial dilution and plate counting on Mueller-Hinton agar plates.

**Table 1:** **Antimicrobial resistant profiles of the *S. aureus *strains used in this study**

**Antibiotic compounds**	***S. aureus *Cowan I ATCC 12598**	***S. aureus *Subspezies Rosenbach ATCC 49230**
**Tigecyclin**	**S**	**S**
**Gentamicin**	**S**	**S**
**Rifampicin**	**S**	**S**

The *S. aureus* strains Cowan I ATCC 12598 and *S. aureus* Subspezies Rosenbach ATCC 49230 were tested for their sensitivity towards antimicrobial compounds used in this study by Vitek-2 (bioMérieux). The minimal inhibitory concentrations (MHK) were interpretated according to the guidelines of EUCAST and CLSI 2012 D.

### Biomechanics

The investigation of the cure time [min] of PMMA-bone cement loaded with tigecyclin versus customary bone cement loaded with gentamicin is performed concerning the ISO 5833-standards. The antibiotics are added with a weight proportion of 1.225%. After 1min the compound of PMMA-bone cement and antibiotic is touched with a dry and clean glove every 15seconds. To determine the compressive strength [MPa] PMMA-bone cement cylinders with a length of 12 mm (± 0,1 mm) and a diameter of 6mm (± 0,1 mm) are produced by the ISO 5833-guidlines with the antibiotics tigecyclin and gentamicin each with a weight proportion of 1.225%. The cylinders are placed into the uniaxial testing machine (Zwick/Roell), which operates a constant compression with a speed of 22 mm/min until the material shows breakage. The compressive strength [MPa] for each test cylinder is then calculated using the following formula: compressive strength [MPa] = (force [N])/(πr^2^), in which the force applied until breakage is divided by the cross-sectional area of the cylinder. The bending strength [MPa] and the bending modulus [MPa] of PMMA-bone cement loaded with tigecyclin or gentamicin (1.225% per weight) are examined conducting ISO 5833-standards with created cement bars with the measure of 75 mm (± 0.1 mm) length, 10 mm (± 0.1 mm) width and 3.3 mm (± 0.1 mm) depth. The test bars are placed into the uniaxial testing machine (Zwick/Roell) and a four-point bending test is accomplished. Operating the crosshead with a speed of 5 mm/min ± 1 mm/min the deflection under the specimen and the applied force are recorded until breakage. The bending strength [MPa] and the bending modulus [MPa] are calculated afterwards taking the defined measurements, the deflection under certain loads and the force at breaking point into account with the formulas: bending strength [MPa] = 3Fa/bh^2^, bending modulus [MPa] = (∆Fa/4fbh^3^) × (3l^2^-4a^2^).

### Statistical analysis

Statistical analysis was performed with the Mann-Whithey-U-test for the results of the infection model. The results of the biomechanical testings besides the bending modulus were statically analysed by the unpaired t-test. Statistical analysis for the bending modulus was performed by the unpaired t-test with Welch correction. A value of * p ≤ 0.05 was consideres siginificant in all cases.

## Results

### Human osteoblast infection model

**Figure 1:**
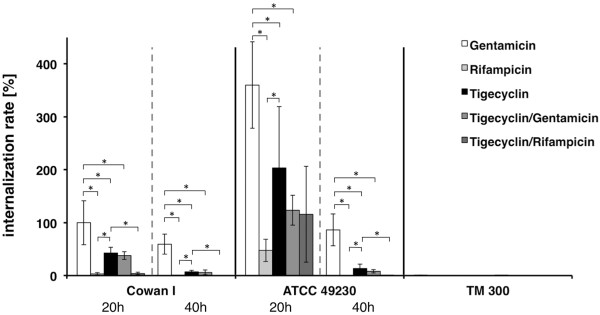
**Relative internalization rate [%] after 20 h versus 40 h.** Primary human osteoblasts were infected with *S*. *aureus* Cowan I ATCC 12598, *S. aureus* Subspezies Rosenbach ATCC 49230 and *S. carnosus* TM 300 (MOI = 100) followed by treatment with antibiotics for 20 h or for 40 h, n = 3. The number of surviving bacteria was determined by plating and is shown in relation to *S. aureus* Cowan I incubated with Gentamicin for 20h (100%). The results are shown as means ± SD of three independent experiments performed in triplicate. * p ≤ 0.05.

Figure 1 shows the relative time-dependent decrease of intracellular bacteria when applying antibiotics to infected osteoblasts. A significant time-dependent difference is noted for the *S*. *aureus* strain Cowan I and 49230 after treating the infected osteoblasts with tigecyclin for 20 h and 40 h. The same time-dependent decreases after 40 h concerning the intracellular bacteria are noticed for all used antibiotics and their combinations. The *S*. *carnosus* strain TM 300, as a non-invasive control, served as a negative control. After incubation for 20h and 40 h cells stimulated with tigecyclin show significant decrease of the intracellular bacteria within the single-use (p = 0.0) and for the combination tigecyclin/gentamicin (p = 0.0) compared to gentamicin. No significant difference can be noted when comparing the combination tigecyclin/gentamicin (p = 0.61-1.0) to tigecyclin. Significant reduction of the intracellular bacteria is investigated for the duration of 20 h and 40 h for the application with rifampicin compared to gentamicin (p = 0.0) and to tigecyclin (p = 0.0). Parallel results are observed for the comparison of the combination rifampicin/tigecyclin with tigecyclin in Cowan I (p = 0.0). No significant difference is found between the combination rifampicin/tigecyclin and rifampicin (p = 0.16-1.0). A significant decrease of intracellular bacteria can be observed after 40 h incubation with tigecyclin compared to 20 h incubation.

### Biomechanics

**Figure 2:**
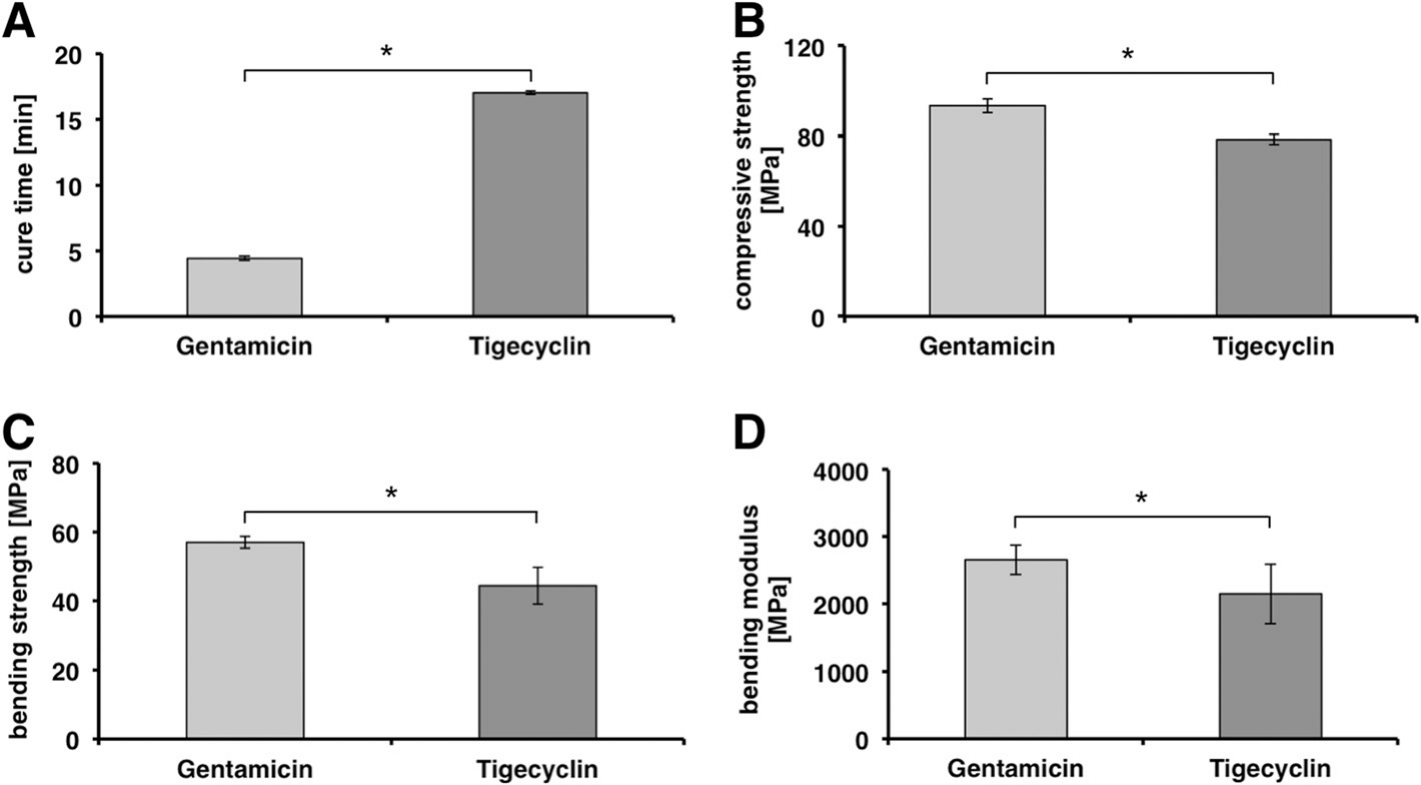
**Demonstration of the biomechanical results. A** cure time [min], **B** compressive strength [MPa], **C** bending strength [MPa], **D** bending modulus [MPa]. The investigation of the biochemical characteristics shows a significant change after adding 1.225% of tigecyclin per weight to PMMA-bone cement compared to customary products with gentamicin. **A** shows the average cure time [min] of the 2 investigation groups. The average compressive strength [MPa] of the 2 different groups is shown in **B**. **C** shows the bending strength [MPa] and **D** the bending modulus [MPa] of the 2 investigation groups. The results are shown as means ± SD of three independent experiments in case of the cure time and six independent experiments for the compressive strength, bending strength and bending modulus. * p ≤ 0.05.

The investigation of the biochemical characteristics shows a significant change after adding 1.225% of tigecyclin per weight to PMMA-bone cement compared to customary products with gentamicin. Figure 2 shows the average cure time [min] of the 2 investigation groups. The average cure time of tigecyclin-loaded bone cement is 17min and therewith significantly (p < 0.0001) extended compared to the customary gentamicin-loaded bone cement. This investigation group only requires 4,42 min for the curing process. The average compressive strength [MPa] of the 2 different groups is shown in Figure 2. The average compressive strength for tigecyclin loaded PMMA-bone cement is 78.4 MPa and 93.41 Mpa for PMMA-bone cement loaded with gentamicin. The average maximum compressive strength in both investigation groups is well exceeding to the required minimum strength of 70 MPa by the ISO, although it is significantly higher for gentamicin (p = 0.0001). Figure 2 also shows the bending strength [MPa] and the bending modulus [MPa] of the 2 investigation groups. The average bending strength for PMMA-bone cement loaded with tigecyclin is 44.30 MPa and is below the ISO required minimum bending strength of 50 MPa. PMMA-bone cement loaded with gentamicin shows a sufficient strength of 56.93 MPa with a significant difference (p = 0.0408). The average bending modulus values are well above the required ISO minimum value of 1800 MPa. The bending modulus amounts 2149.43 MPa to PMMA-bone cement loaded with tigecyclin and 2655.15 MPa to PMMA-bone cement loaded with gentamicin, which points out a significance of p = 0.0387.

## Discussion

The in vitro model of an osteomyelitis displays that the effectiveness of the novel antibiotic tigecyclin has to be ranked between gentamicin and rifampicin after incubation of 20 h and 40 h. The effectivness of rifampicin compared to gentamicin can be confirmed by in vitro and in vivo studies done in 2006 and 2009, in which a reduction of intracellular bacteria after treatment with rifampicin could be shown [34]. In 2008 and 2009 the synergistical effect of tigecyclin to rifampicin and gentamicin against *S*.*aureus* could be displayed in vivo and in vitro as a potential combined therapy against multiresistant bacteria [30,35]. The synergistical effect of tigecyclin to gentamicin was confirmed in our study, whereas the additive effect of tigecyclin to rifampicin could not be shown. It is known that gentamicin has good activity in the extracellular space, whereas an additive effect to the effectiveness of tigecyclin cannot be assigned by our study. However, it remains uncertain if our results can be transferred to in vivo conditions as *S*. *aureus* quickly developes resistances [2]. It would be significant to search out if this has validity for tigecyclin as well. A combination of tigecyclin and rifampicin might be recommendable to prevent resistances. Yin et al. show a complete bacterial eradication after a combined treatment with tigecyclin s.c. and rifampicin p.o. in an in vivo MRSA-osteomyelitis-model [36]. Furthermore, it has to be examined if higher doses of tigecyclin would on the one hand reduce the intracellular bacteria completly and on the other hand would induce the SCV-synthesis, which displays a handicap for antibiotic therapy. 1995 Henry and Galloway showed that PMMA-bone cement loaded with antibiotics is an effective therapy for bone or soft tissue infections as it is possible to reach high antibiotic levels locally without their unwanted side effects [13]. Since 2003 gentamicin is added to PMMA-bone cement as the standard antibiotic substance with a broad antibacterial effectiveness, a low influence on biomechanical properties of bone cement and a good release out of bone cement [12]. Despite the study results it is doubtfull if tigecyclin is a potential alternative to gentamicin for the local use in bone cement. Here the biomechanical investigation of tigecyclin shows a reduction of the stability of Palacos ®-bone cement compared to Palacos ®-bone cement loaded with gentamicin. It is known that bone cement loaded with gentamicin or rifampicin shows a good local effectiveness with reduction of implant-associated bacteria and reduction of biofilm synthesis [19,21,37]. Similar results are not yet exciting for tigecyclin [37], which would be important to consider tigecyclin as an alternative to gentamicin in clinical daily routine. With the help of different models the good release of gentamicin out of bone cement could be displayed [12,38], but it is still uncertain if a constant release of gentamicin induces the synthesis of resistances or not [12]. Bone cement loaded with rifampicin reveals a bimodal elution kinetic with a fast, high concentrated initial release follwed by a slow, low concentrated release [18]. Comparable studies concerning the release of tigecyclin out of bone cement are missing. In actual studies there are only few resistances against tigecyclin listed and it is known as an effective therapy against MRSA-infections and biofilm associated bacteria [25,26,28,39]. Furthermore tigecyclin reduces the expression of some important virulence factors of *S*. *aureus*[40]. 91% of multiresistant *S*. *epidermidis* are sensitive against tigecyclin [4]. Redvold et al. demonstrate the good bone absorption of tigecyclin after intravenous application [27]. On the other hand there is less clinical data exciting concerning this novel antibiotic substance associated with safety questions [41]. In addition Rose and Poppens demonstrate that tigecyclin does not reduce biofilm-associated *S*. *aureus*[42].

## Conclusions

In conclusion there is inconsistent result concerning tigecyclin. Tigecyclin shows effective intracellular activity against *S*. *aureus* with negative influence on the biomechanical stability of Palacos ®-bone cement. In total tigecyclin seems to be a possible therapy option or alternative in the systemic treatment of osteomyelitis because of its good intracellular effectiveness. The local application of tigecyclin has to be evaluated individually because of less biomechanical stability of bone cement.

## Competing interests

This study was funded by the interdisciplinary center for clinical research, Muenster (IZKF, C.K.).

## Authors’ contributions

CK is the corresponding author, carried out the human osteoblast infection model, participated in the biomechanical study and designed the manuscript. MR participated in the design of the study and gave final approval of the version to be published. SR carried out main parts of the biomechanical study. NT made contributions to acquisition of date, analysis and interpretation of data. Furthermore she conceived the human osteoblast infection model. BL has given final approval to the human osteoblast infection model and participated in the interpretation of data. TF conceived of the study and its design. He participated in the design of the manuscript and gave final approval of the version to be published. All authors read and approved the final manuscript.

## Pre-publication history

The pre-publication history for this paper can be accessed here:

http://www.biomedcentral.com/1471-2334/13/267/prepub
